# Technology-Aided Programs to Support Positive Verbal and Physical Engagement in Persons with Moderate or Severe Alzheimer’s Disease

**DOI:** 10.3389/fnagi.2016.00087

**Published:** 2016-04-21

**Authors:** Giulio E. Lancioni, Nirbhay N. Singh, Mark F. O’Reilly, Jeff Sigafoos, Fiora D’Amico, Caterina Renna, Katia Pinto

**Affiliations:** ^1^Department of Neuroscience and Sense Organs, University of BariBari, Italy; ^2^Medical College of Georgia, Augusta UniversityAugusta, GA, USA; ^3^Department of Special Education, University of Texas at AustinAustin, TX, USA; ^4^School of Education, Victoria University of WellingtonWellington, New Zealand; ^5^S. Raffaele Medical Care CenterAlberobello, Italy; ^6^Domus Maxima Care CenterCasamassima, Italy; ^7^Alzheimer CenterBisceglie, Italy

**Keywords:** Alzheimer’s disease, reminiscence, computer-aided program, visual cues, reminders, approval, physical exercise, arm raising

## Abstract

Pilot studies using technology-aided programs to promote verbal reminiscence and mild physical activity (i.e., positive forms of engagement) in persons with moderate or severe Alzheimer’s disease have provided promising results (Lancioni et al., 2015a,b). The present two studies were aimed at upgrading and/or extending the assessment of those programs. Specifically, Study 1 upgraded the program for verbal reminiscence and assessed it with eight new participants. The upgraded version automatically monitored the participants’ verbal behavior during the sessions, in which photos and brief videos were used to foster verbal reminiscence. Monitoring allowed computer approval and reminders to be consistent with the participants’ behavior. Study 2 extended the assessment of the program for promoting mild physical activity with 10 new participants for whom arm-raising responses were targeted. The results of Study 1 showed that the participants’ mean percentages of intervals with verbal engagement/reminiscence were below 10 during baseline and control sessions and between above 50 and nearly 80 during the intervention. The results of Study 2 showed that the mean frequencies of arm-raising responses were about or below four and between about 10 and 19 per session during the baseline and the intervention, respectively. The general implications of the aforementioned results and the need for new research in the area were discussed.

## Introduction

Alzheimer’s disease is a neurodegenerative disorder that progressively undermines the intellectual and physical functioning of the persons affected, with an increasing deterioration of their general performance (i.e., from their use of time and money to their management of daily activities, social and verbal behavior and mobility/ambulation; De Leo et al., 2011; Melrose et al., 2011; Ambrose, 2012; Bernick et al., 2012; Soto et al., 2012; Sikkes et al., 2013). At present, it is not possible to prevent the occurrence of the disease or to cure it effectively (i.e., to eliminate or minimize its effects; Spalletta et al., 2012; Wilson et al., 2012; Perri et al., 2014). Nonetheless, pharmacological and behavioral intervention strategies exist that may have some impact in slowing down the deterioration process, support adaptive and motor performance, and improve social appearance and mood/participation (Giordano et al., 2010; Ferrero-Arias et al., 2011; Bharwani et al., 2012; Boller et al., 2012; Kim et al., 2012; de Vries, 2013; Schecker et al., 2013; Berk et al., 2014; Kurz and Grimmer, 2014; Tifratene et al., 2014).

Behavioral intervention strategies employed for persons in the moderate and severe stages of the disease have been directed at supporting, among others, daily activities, verbal reminiscence, and mild physical exercise (Lancioni et al., 2010, 2012; Crete-Nishihata et al., 2012; Serrani Azcurra, 2012; Subramaniam and Woods, 2012; Cavallo et al., 2013; Lazar et al., 2014; Wingbermuehle et al., 2014; Phillips et al., 2015). In each of these intervention areas, technology-aided programs were developed and assessed with promising results. For example, Lancioni et al. (2014b, 2015a) reported encouraging results with a technology-aided program set up to foster positive verbal engagement/reminiscence in persons with moderate Alzheimer’s disease. The program relied on the presentation of: (a) images of relevant people/events together with requests to talk about them or a virtual partner asking questions; (b) approval for verbal engagement; and (c) reminders (prompts) to seek new images/questions (see also Yasuda et al., 2009; Kuwahara et al., 2010; Lazar et al., 2014). Different sets of images and questions were used across sessions. Similarly, Lancioni et al. (2015b) reported a positive outcome with a technology-aided program aimed at supporting mild physical exercise (i.e., arm raising or leg-foot movement) in persons with advanced Alzheimer’s disease, who had lost their ambulation skills and were sedentary and largely inactive and detached. The program included: (a) preferred stimulation contingent on the target response and (b) verbal reminders in case of no responding.

Although the results obtained with the aforementioned technology-aided programs were quite promising, the evidence available is limited. In fact, a total of 22 participants were involved in the two studies targeting positive verbal engagement/reminiscence, and six participants were involved in the study targeting mild physical exercise (i.e., three used arm raising and three leg-foot movement). Given this situation, additional research efforts seem to be warranted to determine the strength of the data available and conceivably, investigate upgrades of the programs so as to make them more functional and/or respectful of the participants (Barlow et al., 2009; Kazdin, 2011; Godwin et al., 2013; Makel and Plucker, 2014).

The present studies were two such research efforts. Study 1 upgraded the technology-aided program for promoting positive verbal reminiscence and assessed such program with eight new participants (thus representing a systematic replication study; see Kazdin, 2011). The new program version (a) presented images of relevant people/events with requests to talk about them and approval and reminders to seek new images, and (b) monitored the participants’ verbal behavior via a voice-detecting sensor (Lancioni et al., 2014a). Such monitoring allowed computer approval and reminders to be consistent with the participants’ behavior (i.e., to occur during and in the absence of verbal engagement, respectively, rather than at preset intervals; see Lancioni et al., 2014b, 2015a). The view was that the use of monitoring would preserve or even strengthen the effectiveness of the original program in fostering positive verbal engagement while increasing the level of respect for the participants and their self-determination (Pierce and Cheney, 2008). Study 2 sought to extend the evidence available on the effectiveness of the technology-aided program designed for promoting mild physical exercise (Lancioni et al., 2015b). Specifically, Study 2 implemented the program with 10 new participants who were involved in an arm-raising exercise, with the aim of determining whether preliminary promising results with this form of exercise could be confirmed in a carefully designed, direct replication effort (Kazdin, 2011).

## Study 1

### Materials and Methods

#### Participants

The eight participants involved in Study 1 included five females and three males, aged 73–96 (*M* = 82) years, and had a diagnosis of moderate Alzheimer’s disease with reported scores on the Mini Mental State Examination (Folstein et al., 1975) ranging from 12 to 18 (*M* = 15; see Table 1). The participants tended to be passive and silent, but were capable of responding to verbal questions and reminders, and of watching photos and videos and talking about them. That is, they possessed the skills needed to use the computer program, seemed comfortable with it, and were at ease wearing the aforementioned voice-detecting sensor, which involved a small throat microphone kept at their larynx with a neckband (see Lancioni et al., 2014a).

**Table 1:** **Participants’ characteristics (Study 1)**.

Participant	Sex	Age	MMSE
1	F	81	18
2	M	76	14
3	F	80	16
4	F	89	14
5	M	81	12
6	F	96	17
7	M	73	13
8	F	78	14

*MMSE stands for Mini Mental State Examination*.

The participants attended centers for persons with Alzheimer’s disease and other dementias, in which they were involved in self-care (e.g., grooming) and leisure (e.g., listening to music) activities. They could also have fairly long periods of inactivity, with only marginal staff supervision. Those periods of inactivity and general silence were considered detrimental and an intervention strategy to counteract them was viewed as desirable. The use of a simple computer-aided program to help the participants reminisce events of their life and increase their positive verbal engagement (as pursued in this study) seemed a practical example of such strategy. Participants had expressed their willingness to be involved in the program and staff and families supported it (Lancioni et al., 2015a). Participants had not been asked to sign a formal consent for their involvement in the study, as they were not considered capable of reading a complex text and/or writing. Families had however provided a written informed consent for such an involvement. The study had been approved by the ethics committee of the Alzheimer Association, Bari, Italy.

#### Technology

The technology involved a computer system with screen and sound amplifier, a pressure microswitch, a voice-detecting sensor with throat microphone, and basic software. During intervention sessions, the participant had the pressure microswitch (a push button) in front of him or her and the voice-detecting sensor’s microphone (which was activated by any audible vocal emission and was unaffected by environmental noise), kept at his or her larynx with a neckband. The participant sat in front of the computer screen, which showed photos or 5-s video clips of relevant people (including him- or herself) and special places and/or community and family events, such as wedding celebrations (Lancioni et al., 2015a). The computer provided a brief verbal description of the photos and the videos (the last frame of which remained on view like the photos) and asked the participant to talk about them. At intervals of 25–50 s, the computer produced approving vocal sounds/words if the participant was talking. A reminder to press the push button and talk more occurred after 10–20 s of participant’s silence/passivity (i.e., no activation of the voice sensor or push button). Activation of the push button caused the computer to present a new photo or video (i.e., as described above). Failure to activate the push button and the voice sensor led the computer to provide additional reminders. About 25–40 photos and videos were selected for the single participants with the help of staff and families for use across sessions.

#### Setting, Sessions, and Data Recording

Baseline, intervention, and control sessions were carried out in the centers that the participants attended. Sessions lasted 5 min. Typically, two to four baseline or intervention sessions occurred per day per participant (i.e., sessions were carried out on an individual basis). Control sessions were scattered throughout the intervention phase (see below). Data recording concerned: verbal engagement/reminiscence, microswitch activations, and computer reminders (during intervention sessions), the first two of these measures (during baseline sessions), or the first one of these measures (during control sessions). Microswitch activations and computer reminders were recorded by the computer system in terms of frequency per session. Verbal engagement/reminiscence was recorded by research assistants, who used a time sampling procedure with 10-s intervals, and checked reliability in about 25% of the sessions (Kazdin, 2001). The percentages of interrater agreement in those sessions (computed by dividing session intervals with agreement by total number of session intervals and multiplying by 100) were within the 80–100 range, with means above 90 for all participants.

#### Experimental Conditions and Data Analysis

The program was carried out according to a non-concurrent multiple baseline design across participants (Barlow et al., 2009). The baseline phase included two or four sessions per participant. The intervention phase included 98 to 147 (*M* = 126) sessions. Parallel to the intervention phase, the participants received 9–20 (*M* = 14) control sessions. A nonparametric (Kolmogorov-Smirnov) test was used to assess differences in the participants’ levels of verbal engagement during the intervention sessions (with the technology) and the baseline and control sessions (in which the technology was inoperative or absent; Siegel and Castellan, 1988). Such a test was employed because of the differing numbers of sessions available to the participants during the study periods (i.e., in line with the research design applied for the study; see Barlow et al., 2009; Kazdin, 2011).

##### Baseline

During the baseline sessions, the participant sat in front of the computer screen, which was dark, and had the pressure microswitch and voice sensor whose activations did not produce any effects.

##### Intervention

During the intervention sessions, the participant was provided with the computer, pressure microswitch and voice sensor, which worked as described in the Technology section. Prior to the start of the intervention phase, each participant received five to seven practice sessions during which the research assistant helped him or her familiarize with procedural conditions and verbal reminiscence.

##### Control

During the control sessions (which epitomized periods of general inactivity; see Participants section), no technology was available. The participants sat with other persons with dementia attending the same context, with only marginal staff supervision.

### Results

Table 2 reports the participants’ mean percentages (and percentage ranges) of verbal engagement intervals per session during the baseline and intervention phases of the study. During the baseline sessions, the mean percentages of intervals with verbal engagement/reminiscence were below 10 for all participants. During the intervention sessions, the mean percentages of intervals with verbal engagement/reminiscence ranged from above 50 (Participant 7) to nearly 80 (Participant 2), with the overall mean exceeding 65. The mean frequencies of microswitch activations were (near) zero during the baseline and varied from about two and a half to about six and a half per session (with an overall mean across participants of about four) during the intervention. The mean frequencies of computer reminders (available only during the intervention sessions) varied from below one to about three per session, with an overall mean across participants of about two. During the control sessions, the participants’ mean percentages of intervals with verbal engagement/reminiscence were similar to those recorded during the baseline (i.e., below six). The Kolmogorov-Smirnov test showed that the differences between the participants’ levels of verbal engagement during the intervention sessions and during the baseline and control sessions combined were statistically significant (*p* < 0.01; Siegel and Castellan, 1988).

**Table 2:** **Number of sessions, mean percentages of verbal engagement intervals per session, and percentage ranges during baseline and intervention (Study 1)**.

	Baseline			Intervention		
Participant	Sessions	Means	Ranges	Sessions	Means	Ranges
1	2	0	0–0	134	65	33–83
2	2	0	0–0	147	79	33–90
3	2	8	7–10	132	72	37–87
4	4	2	0–7	115	74	43–90
5	2	0	0–0	98	68	37–80
6	4	8	3–17	139	60	30–77
7	4	2	0–7	105	53	23–73
8	4	1	0–3	141	63	30–83

*Percentages are rounded to the nearest full number value*.

## Study 2

### Materials and Methods

#### Participants

The 10 participants involved in Study 2 included five females and five males, aged 67–95 (*M* = 82) years. They were at the moderate or severe level of Alzheimer’s disease, with reported scores on the Mini Mental State Examination (Folstein et al., 1975) of 7 to 14 (*M* = 9; see Table 3), and attended centers for persons with Alzheimer’s disease and other dementias. They were sedentary and generally passive, but: (a) could perform arm-raising responses deemed beneficial for their physical condition (Rolland et al., 2008; Eggermont et al., 2010; Hoffmann et al., 2013); (b) responded to computer-emitted verbal reminders concerning those responses; and (c) were interested in environmental stimulation events, such as music and videos. Staff and families supported the use of a technology-aided program for fostering arm movements through preferred stimulation for those movements and reminders. Participants seemed keen to be involved in the program, but were not asked to sign a consent form (see “Study 1” Section). The participants’ families had provided a written informed consent for the study, which had been approved by the same ethics committee as Study 1.

**Table 3:** **Participants’ characteristics (Study 2)**.

Participant	Sex	Age	MMSE
1	F	90	11
2	F	67	8
3	F	88	7
4	M	78	10
5	M	76	11
6	F	84	14
7	M	90	10
8	M	68	9
9	M	84	7
10	F	95	8

*MMSE stands for Mini Mental State Examination*.

#### Sessions, Responses, Technology, and Stimulation

Sessions lasted 5 min and were typically carried out 2–4 times a day, in the centers that the participants attended. Arm raising consisted of the participants moving one or both arms upward. The technology included a microswitch, a computer with sound amplifier, and basic software. The microswitch consisted of a tilt device or a combination of two such devices fixed to the participant’s arms (Lancioni et al., 2015b). The computer’s use during baseline was limited to recording the arm-raising responses. During the intervention, the computer: (a) delivered a 10-s stimulation after each arm-raising response; (b) presented a verbal reminder to raise the arms after periods of about 15 s of no response from the start of the session or from the end of a stimulation period; and (c) recorded the arm-raising responses and the reminders (Lancioni et al., 2015b).

The stimulation entailed 10-s periods of popular songs, religious chants, and prayers or videos of friends and activities, which were considered preferred for the participants based on staff reports and stimulus preference screening. Screening included a minimum of 10 nonconsecutive presentations of brief segments of various stimuli. A stimulus was used during the study if, during screening, two research assistants scored it as effective in producing positive reactions (e.g., orienting, smiling, or comments) in 60% or more of the presentations.

#### Experimental Conditions and Data Analysis

The study was carried out according to a non-concurrent multiple baseline design across participants (Barlow et al., 2009). That is, five participants had two or three baseline sessions while the other five participants had four or five baseline sessions. The baseline was followed by an intervention phase, which included 93 to 140 (*M* = 119) sessions for eight participants. The other two participants (i.e., Participants 7 and 9) had only 63 and 61 intervention sessions due to departure from the care center and to health problems, respectively. During the sessions, the participants sat in a wheelchair or normal chair. Prior to the start of the sessions, the research assistants guided them to perform the target response. The Kolmogorov-Smirnov test (Siegel and Castellan, 1988) was used to assess differences between the participants’ frequencies of arm-raising responses during the intervention sessions and during the baseline sessions (see “Study 1” Section).

##### Baseline

During the baseline sessions, the technology was available but no stimulation or reminders occurred.

##### Intervention

During the intervention sessions, the technology was in use and worked (i.e., provided stimulation and reminders) as described above. The intervention sessions were preceded by five to eight practice/familiarization sessions (see “Study 1” Section).

### Results

Table 4 reports the participants’ mean frequencies (and frequency ranges) of arm-raising responses per session during the baseline and the intervention. During the baseline, the participants’ mean frequencies of arm-raising responses per session were between zero and four. During the intervention, the mean frequencies of arm-raising responses per session varied from about 10 (Participants 5 and 10) to about 19 (Participant 6), with an overall mean across participants of about 14. The mean frequencies of computer reminders varied from below one to about nine per session, with an overall mean across participants of near four. The Kolmogorov-Smirnov test showed that the differences between the baseline and the intervention response frequencies were statistically significant for all participants with *p* < 0.01 (Participants 1–8) and *p* < 0.05 (Participants 9 and 10; Siegel and Castellan, 1988). The latter *p* value was due to the fact that Participants 9 and 10 had only two baseline sessions.

**Table 4:** **Number of sessions, mean frequencies of arm-raising responses per session, and frequency ranges during baseline and intervention (Study 2)**.

	Baseline			Intervention		
Participant	Sessions	Means	Ranges	Sessions	Means	Ranges
1	4	3	0–8	104	15	9–22
2	5	4	3–5	112	14	9–20
3	5	1	0–3	138	17	11–21
4	3	1	1–1	109	10	5–15
5	5	2	1–4	119	11	8–16
6	5	2	0–4	140	19	15–23
7	3	1	0–2	63	13	6–20
8	3	2	1–6	136	18	14–23
9	2	0	0–0	61	12	7–18
10	2	0	0–0	93	10	9–12

*Frequencies are rounded to the nearest full number value*.

## Discussion

Studies 1 and 2 provided new evidence on the usability and effectiveness of computer-aided programs for promoting: (a) positive verbal reminiscence in persons with moderate Alzheimer’s disease and (b) mild physical exercise in persons with low moderate or severe levels of Alzheimer’s disease, respectively (Astell et al., 2010a,b; Lancioni et al., 2014b, 2015a,b; Lazar et al., 2014; Makel and Plucker, 2014). Study 1 also showed a relatively simple way of upgrading the technology for the verbal reminiscence program compared to the versions originally employed (Lancioni et al., 2015a). In light of this upgrading and the results obtained in the two studies, a number of considerations may be in order.

First, the technology upgrading operated in Study 1, that is, the inclusion of a sensor to monitor the participant’s verbal behavior did not alter the basic principles/components of the original program (i.e., presentation of images of relevant persons, events or places, requests to talk about them, approval, and reminders; Lancioni et al., 2015a). In reality, the upgrading served to make the program more consistent with (and respectful of) the participant’s behavior by ensuring that: (a) positive comments would be contingent on the participant’s verbal engagement and (b) reminders would not occur while the participant was still talking (Noguchi et al., 2013; Robert et al., 2013; König et al., 2015).

Second, preservation of all the basic principles/components of the original program for supporting verbal reminiscence engagement was considered a sufficient condition to expect the new, upgraded program to be at least as effective as the original one. Consistent with this assumption, we (a) did not seek to compare the two program versions and (b) treated the data obtained with the upgraded version as an important expansion of the original evidence about the possibility of supporting verbal reminiscence through a technology-aided program (Kazdin, 2001, 2011; Kennedy, 2005; Barlow et al., 2009). It would be theoretically and practically relevant for future research to investigate whether the impact of such positive verbal engagement can extend beyond the constructive/satisfactory experience of the intervention situation (i.e., beyond the sessions’ time and context), and represent a partial and/or temporary form of protection against the inevitable degenerative process caused by the disease (Buchhold et al., 2007; Huang et al., 2009; Chiang et al., 2010; Saczynski et al., 2014; Freret et al., 2015; Bechard et al., 2016; Fuchs et al., 2016).

Third, the data of Study 2 add strength to the early evidence on the importance of a computer-aided program for helping persons in the later stages of the Alzheimer’s disease engage in mild physical exercise on their own, presumably: (a) motivated by the stimulation available for their responses and (b) encouraged by the reminders occurring in case of response discontinuity (Kazdin, 2001; Pierce and Cheney, 2008; Letts et al., 2011; Catania, 2012; Halpern, 2012). The same program could also be considered useful for supporting other forms of arm and leg responses and thus extending and varying the exercise opportunities and possibly increasing the beneficial consequences for the participants (e.g., protecting or improving their mobility and mood; Christofoletti et al., 2011; Intlekofer and Cotman, 2013; Farina et al., 2014; Lancioni et al., 2015b; Paillard et al., 2015; Phillips et al., 2015; Bechard et al., 2016). In the absence of such a program, persons like those involved in Study 2 would be generally passive and sedentary (i.e., in a condition entailing reduced levels of direct stimulation) with only limited chances of relying on staff’s help. In fact, staff might only rarely have sufficient time to ensure specific, individualized delivery of contingent stimulation and reminders (Williams and Tappen, 2008; Brown et al., 2009; Friedman et al., 2009; Lancioni et al., 2009a,b; Wood et al., 2009; Pilotto et al., 2011; Rimmer and Marques, 2012; Perales et al., 2013).

In conclusion, the results of the two studies confirm that relatively simple technology-aided programs might be used profitably for supporting independent (i.e., computer-mediated) verbal engagement/reminiscence and mild physical exercise in persons with Alzheimer’s disease. In spite of these encouraging results, some caution may be required in making definite statements about the impact and practical implications of the studies, given that the numbers of participants were fairly small and the research designs and data analyses were fairly unsophisticated. New research efforts are needed to: (a) extend the assessment of the programs with additional participants, thus rectifying a limitation of the present studies and of previous ones in the area; (b) investigate the potential of reminiscence and physical exercise for granting the participants some level of protection against (an opportunity to slow down) their inevitable decline; and (c) seek new technology solutions and additional exercise responses to improve the programs’ applicability and friendliness (Kennedy, 2005; Barlow et al., 2009; Subramaniam and Woods, 2012; Hannan, 2014; Saczynski et al., 2014; Wingbermuehle et al., 2014; Bezzina et al., 2015; Freret et al., 2015; Mulfari et al., 2015; Phillips et al., 2015; Bechard et al., 2016; Fuchs et al., 2016). Evaluating the participants’ comfortableness with the technology packages adopted and gathering staff and families’ opinions about such packages would be essential to design adaptations of the present programs and develop new, successful alternative solutions for applied contexts (Callahan et al., 2008; de Joode et al., 2012, 2013; Robert et al., 2013; Meiland et al., 2014; König et al., 2015).

## Author Contributions

NNS, MFO’R, JS and FD’A: Work conception, data acquisition/analysis, and manuscript editing (plus final approval and accountability). CR and KP: Data acquisition/analysis, and manuscript editing (plus final approval and accountability). All authors listed, have made substantial, direct and intellectual contribution to the work, and approved it for publication.

## Conflict of Interest Statement

The authors declare that the research was conducted in the absence of any commercial or financial relationships that could be construed as a potential conflict of interest.
